# Supplementary material for: Ultra-large single crystals by abnormal grain growth
Source: Nat Commun. 2017 Aug 25;8:354. doi: 10.1038/s41467-017-00383-0 (PMC5572478; doi:10.1038/s41467-017-00383-0)
Supplement: Supplementary file 2 — Supplementary Information [file 41467_2017_383_MOESM2_ESM.pdf]

File Name: Peer Review File

File Name: Supplementary Information
Description: Supplementary Figures, Supplementary Discussion, Supplementary References.

File Name: Supplementary Movie 1
Description: Superelasticity of Cu-Al-Mn single crystal. Bending test of Cu-Al-Mn single crystal bar 16 mm in diameter and 680 mm in length produced by cyclic heat treatment. Superelastic response with almost no residual strain is observed.

File Name: Supplementary Movie 2
Description: Superelasticity of Cu-Al-Mn polycrystalline alloy. Bending test Cu-Al-Mn polycrystalline alloy bar 16 mm in diameter and 680 mm in length. Only partial superelasticity with noticeable residual strain is observed.

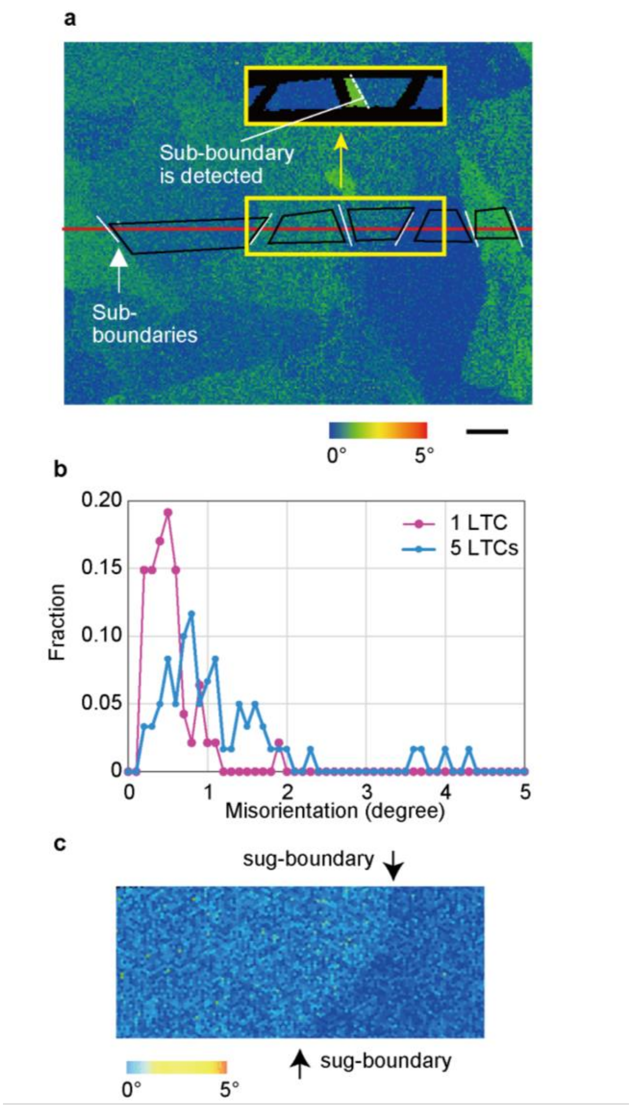

Supplementary Figure 1. Method of determination of misorientation angle between subgrains. (**a**) Step 1: A line is drawn in the grain reference orientation deviation (GROD) map (red line), where a sub-boundary can be roughly identified with a GROD color change. Step 2: The detected subgrain is cut by OIM Analysis software (black line). Step 3: GROD maps for all cut images are recreated, in which a sub-boundary can be precisely detected, as shown in the inset. Thus, all the sub-boundaries measured are identified and then the misorientation of each sub-boundary can be calculated. The scale bar is 20 μm. (**b**) Fraction of misorientation angle between average orientations in each subgrain in one low-temperature cycle (LTC) (Supplementary Fig. 4d) and five LTC (Supplementary Fig. 4e) specimens. Peak is located in lower angle in one LTC specimen, and average misorientations in one LTC and five LTC specimens are 0.46° and 1.12°, respectively. (**c**) GROD map with the minimum misorientation (0.11°) detected in this work.

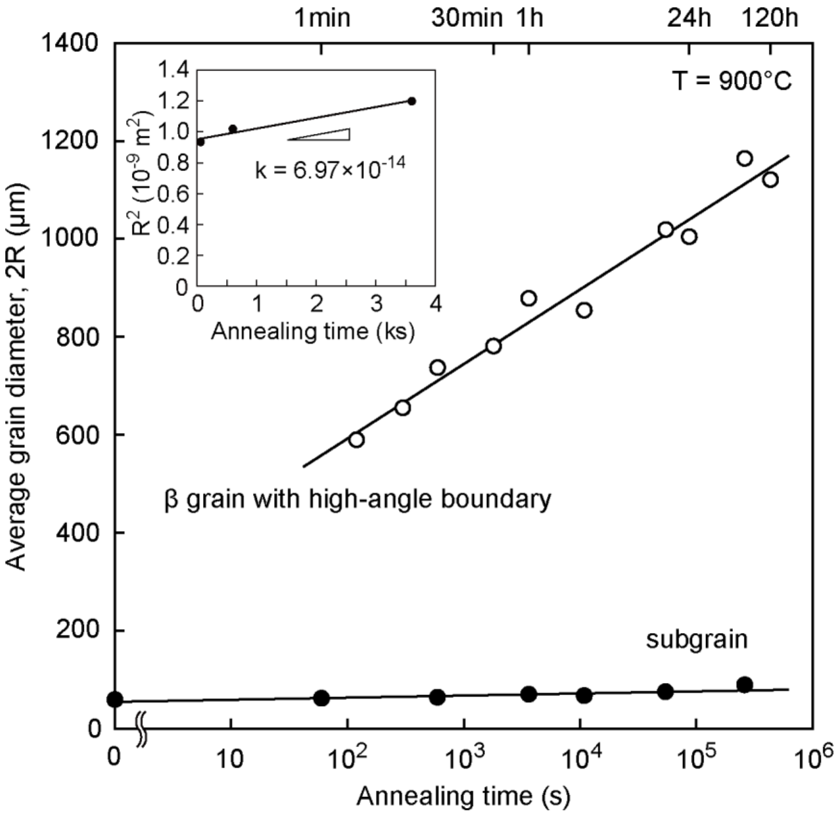

Supplementary Figure 2. Average grain diameter of subgrains at 900ºC as a function of annealing time in Cu-Al-Mn alloy. The subgrains were obtained by cyclic heat treatment (CHT). Compared with the grain growth of the β grain with a high-angle boundary,[1] the growth of subgrains is very slow. The growth rate in normal grain growth (NGG) is expressed by the following equation: $\bar{R}^n - \bar{R}_0^n = kt$, where $\bar{R}$ and $\bar{R}_0$ are average grain radii at annealing time t and that at $t = 0$, respectively, and k is constant. The grain growth exponent, n, is theoretically 2 in a single-phase condition, but n determined in the whole time range was 12 in the present study. The k for $n = 2$ can be determined for annealing time up to 3.6 ks to be 6.97×10^{-14} m^2 s^{-1}, as shown in the inset. The lower growth rate of the subgrains is mainly due to the low mobility of low-angle boundaries.

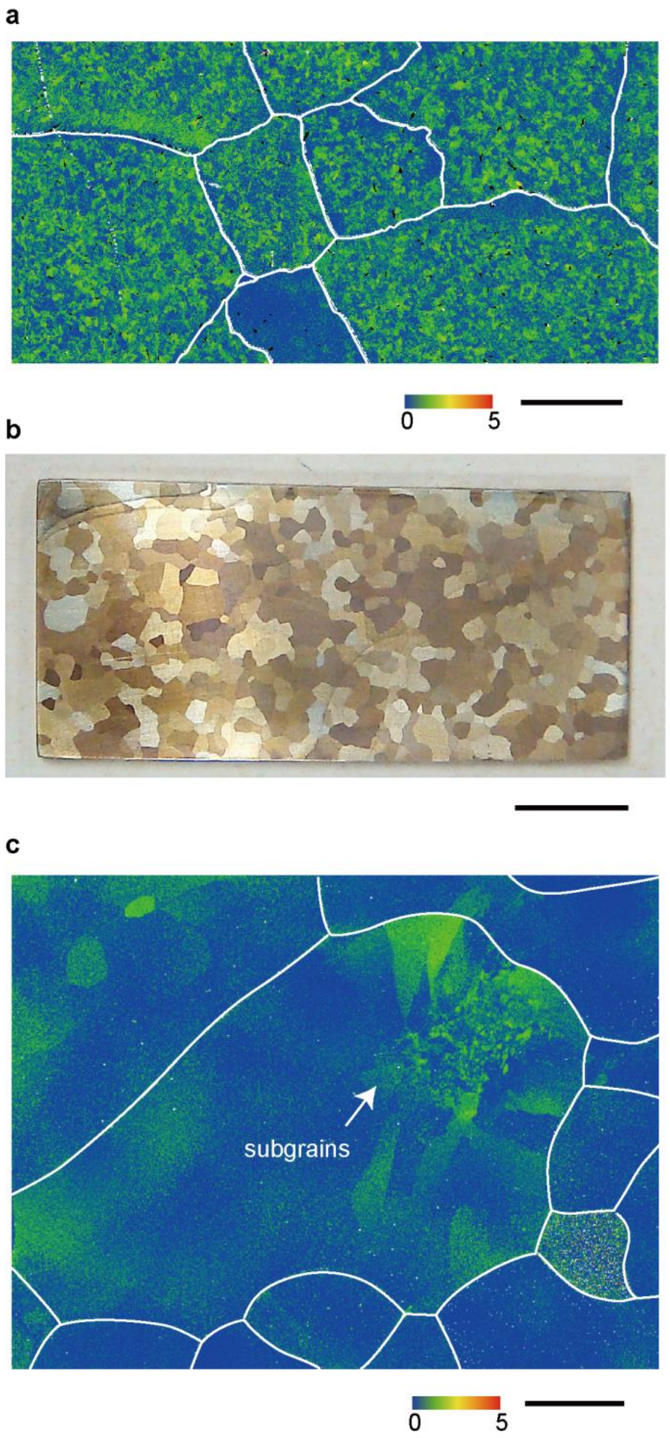

Supplementary Figure 3. Microstructure of Cu-Al-Mn alloy subjected to step heat treatment. (**a**) Grain reference orientation deviation (GROD) mapping of Cu-Al-Mn alloy subjected to isothermal step heat treatments at 900 ºC for 5 min, 500 ºC for 10 min and 800 ºC for 2 min (Supplementary Fig. 4g). Distribution of orientation deviation is more homogeneous. The white lines indicate high-angle boundary. The scale bar is 300 μm. (**b**) Microstructure after heat treatment of Supplementary Fig. 4h. The scale bar is 5 mm. (**c**) GROD mapping of (b). Subgrains do not exist within grains except in the top right part. The scale bar is 300 μm.

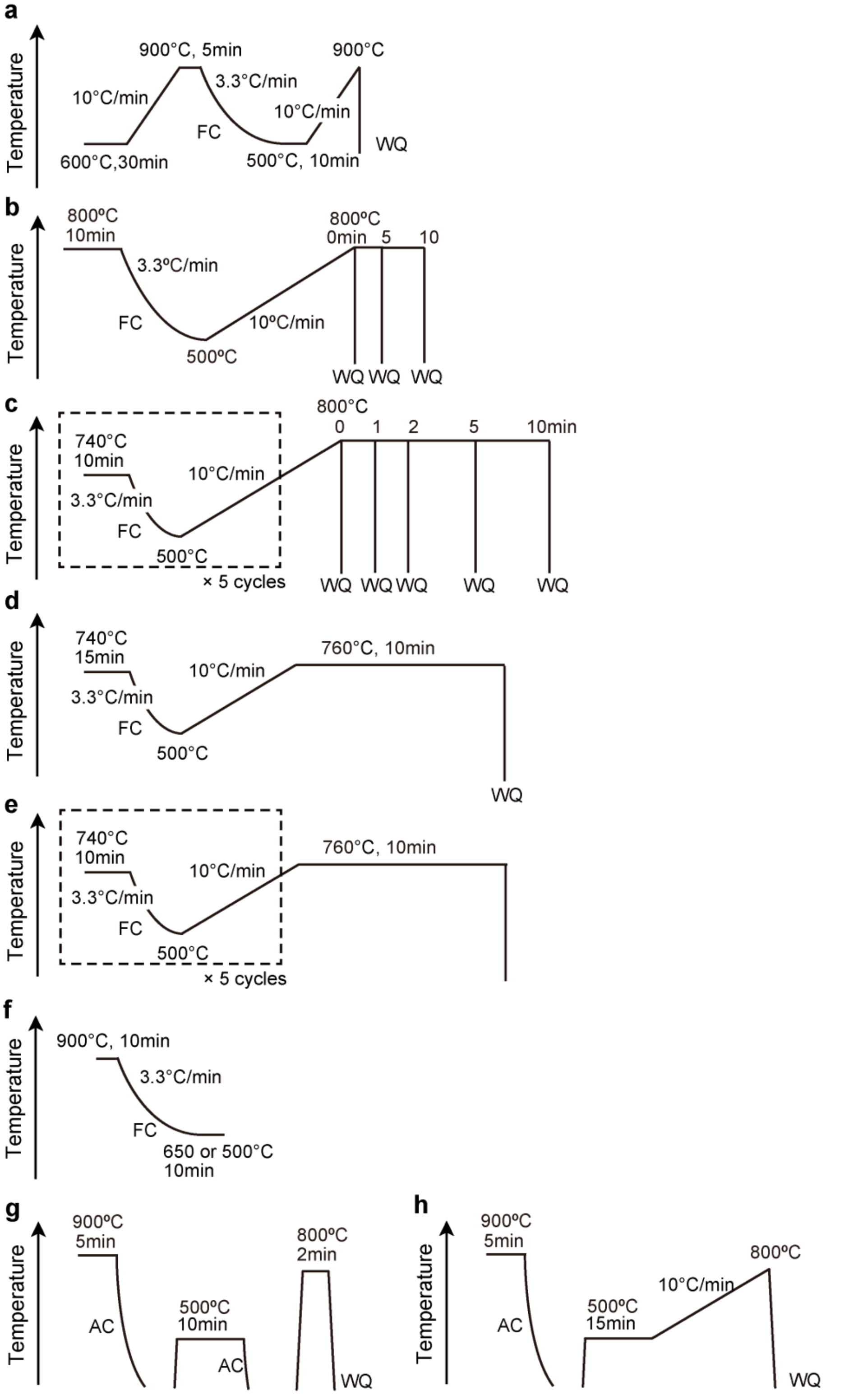

Supplementary Figure 4. Cyclic heat treatment (CHT) of sheet specimens. (**a**) CHT for Fig. 2. (**b**) CHT for Fig. 4a and Fig. 4c. (**c**) CHT for Fig. 4b and Fig. 4c. (**d**) CHT for 1 cycle of Fig. 4d, Fig. 4e and Supplementary Fig. 1. (**e**) CHT for 5 cycles of Fig. 4d, Fig. 4e and Supplementary Fig. 1. (**f**) CHT for Fig. 5. (**g**) CHT for Supplementary Fig. 3a. (**h**) CHT for Supplementary Fig. 3b. (WQ: water quenching, AC: air cooling, FC: furnace cooling.)

Supplementary Discussion

1. Accuracy of misorientation

The error of the electron backscatter diffraction (EBSD) experiments should be considered because the accuracy of the absolute orientation in EBSD is said to be 0.5° to 1°[2, 3]. In order to determine the average orientation of each subgrain, at least 200 points in one subgrain were averaged. The standard error σ_M is given by

$$\sigma_M = \frac{\sigma}{\sqrt{n}} , \qquad (1)$$

where σ is the standard deviation and n is the number of data. We use $\sigma = 1°$ and $n = 200$ as the worst case, and σ_M is calculated to be 0.07°. Therefore, the maximum error of difference between average orientations is 0.14°, and the average θ can be written as 0.46° ± 0.14° and 1.12° ± 0.14° for one low-temperature cycle (LTC) and five LTCs, respectively. It is also important not to miss the sub-boundaries in the grain reference orientation deviation (GROD) mapping. Supplementary Fig. 1c shows the GROD map with the minimum misorientation of 0.11° detected in this study.

2. Grain selection during AGG

It is an important issue whether or not grain selection for abnormal grain growth (AGG) occurs. Every grain boundary receives stored energy for AGG and every grain can grow unless some grain boundaries are pinned by precipitates or have low boundary energy. As seen in the microstructures of Fig. 4a (5 min and 10 min), only a limited number of grains start to grow in finer grained matrix, namely, grain selection and a typical microstructural feature of AGG. It was confirmed in the present case that no special grain-boundary-character distribution develops, in contrast with the usual AGG. We also observed that AGG occurs at temperatures several tens of Kelvin higher than the α solvus temperature.

Careful observation of the microstructure of the normal grains in Fig. 2c reveals that the

orientation deviation is lower in the vicinity of high-angle boundaries and that the density of subgrains around the high-angle boundaries is relatively lower than in the central part of grains. This typical microstructure may be due to formation of a low density zone (LDZ) of α precipitates near grain boundaries in the two-phase temperature region as seen in Fig. 5. Therefore, AGG may not immediately start until the grain boundaries cross over the LDZ of subgrains by normal grain growth (NGG), namely, some "incubation" time for AGG is required. This means that grain selection for AGG occurs due to microstructural inhomogeneity in the initial NGG process. In order to judge this possibility, we prepared a sample with the different microstructure obtained by cyclic heat treatment (CHT) with rapid cooling and heating, which has a relatively thin LDZ of subgrains, as shown in Supplementary Fig. 3a. The specimen slowly heated to the β single phase region at 800 °C after rapid cooling has a relatively small and uniform grain structure, as seen in Supplementary Fig. 3b. It is interesting to note that most grains hardly include subgrains, as shown in Supplementary Fig. 3c, meaning that since AGG occurs simultaneously for many grains, the AGG grains immediately come in contact with one another, resulting in formation of a homogeneous microstructure with relatively small grains. This result means that the width of the LDZ of subgrains, resulting from the LDZ of α precipitates, is one of the most important factors for development of the inhomogeneous AGG and that the slow cooling in the CHT, which yields a coarse precipitation structure, is very important. In this consideration, it should be pointed out that the heating process in the CHT is also very important. The "incubation" time for the grain boundaries to cross over the LDZ of subgrains is obviously related to the dissolution process of the α precipitates. Since being able to easily move in advance of other boundaries with pinning precipitates, some grain boundaries, where α precipitates are resolved early, may be able to cross over the LDZ of subgrains earlier. This means that the slower the CHT heating rate, the more remarkable the microstructural inhomogeneity. Actually, in our experiments, larger abnormal grains were obtained by decreasing both the cooling and heating rates of the CHT.

When a limited number of previously formed abnormal grains (AGs) grow in the surrounding normal grains after the following CHT, the large AGs may initially start to selectively grow by the NGG mode caused by the effect of the curvature of the grain boundary. After the grain boundaries of the AGs cross over the LDZ of subgrains, however, drastic growth by the AGG mode actually starts. Therefore, the same large AGs are always selected as growing grains in further CHTs. Such grain selection, however, may no longer occur in a bamboo structure. Nevertheless, the total number of grains continues to decrease through further CHT due to the disappearance of grain boundaries at the ends of the sample and the unification with neighbouring grain boundaries.

Supplementary References

1. Kusama T, Omori T, Saito T, Ohnuma I, Ishida K, Kainuma R. Two- and Three-Dimensional Grain Growth in the Cu-Al-Mn Shape Memory Alloy. *Materials Transactions* **54**, 2044-2048 (2013).

2. Humphreys FJ, Brough I. High resolution electron backscatter diffraction with a field emission gun scanning electron microscope. *Journal of Microscopy-Oxford* **195**, 6-9 (1999).

3. Humphreys FJ. Quantitative metallography by electron backscattered diffraction. *Journal of Microscopy-Oxford* **195**, 170-185 (1999).
